# The ovarian and uterine responses of Baixadeiro mares to prostaglandin synchronization during the dry and rainy seasons

**DOI:** 10.1590/1984-3143-AR2020-0050

**Published:** 2022-02-04

**Authors:** Luciana Cordeiro Rosa, Eliane Cristina Silva Dias, Renatta Silva Melo, Carla Janaína Rebouças Marques do Rosário, Felipe Lucas Correa Pereira, Luiz Bruno Oliveira Chung, Adriana Raquel de Almeida da Anunciação, Felipe de Jesus Moraes, Fernando Andrade Souza, Ricardo de Macêdo Chaves

**Affiliations:** 1 Laboratório de Reprodução Animal Universidade Estadual do Maranhão São Luís MA Brasil Laboratório de Reprodução Animal, Universidade Estadual do Maranhão, São Luís, MA, Brasil; 2 Faculdade de Medicina Veterinária e Zootecnia Universidade de São Paulo São Paulo SP Brasil Faculdade de Medicina Veterinária e Zootecnia, Universidade de São Paulo, São Paulo, SP, Brasil e Faculdade Maurício de Nassau, São Luís, Brasil.; 3 Laboratório de Reprodução Animal Universidade Federal do Paraná Curitiba PR Brasil Laboratório de Reprodução Animal, Universidade Federal do Paraná, Curitiba, PR, Brasil

**Keywords:** estrus, follicular development, Baixadeiro mares

## Abstract

This study aimed to evaluate the effect of synchronization with prostaglandin F2α in Baixadeiro mares during the rainy and dry seasons. Fourteen mares were synchronized by administering two doses of 1 mL prostaglandin PGF 2α and monitored by rectal palpation and ultrasound for the assessment of follicular development and uterine echotexture. Of this total, nine mares allowed the collection of blood, in which the blood was collected by venipuncture of the jugular vein to determine progesterone (P4) by ELISA. Mares showed no differences (*P* > 0.05) in weight, body score condition (BSC), tone, uterine edema, frequency of ovulation, synchronization interval, estrus, and the total number of follicles between periods. However, there was a difference in large increased follicle diameter (*P* < 0.05) during the dry season. The average concentrations of P4 in mares differed (*P* < 0.05) between the pre- and post-ovulatory phases for both seasons and after ovulation, with higher concentrations in the rainy season. Furthermore, statistical differences in daily light (*P* < 0.05) were observed between the dry and rainy periods. Thus, we conclude that mares from the genetic grouping Baixadeiro showed no reproductive seasonality, though there was a difference in luminosity between the rainy and dry seasons. The treatment with two doses of PGF 2α was effective in synchronizing the mares, promoting the return of estrus in the dry and rainy periods. The mares remaining cyclically active throughout the year provided there were appropriate forage availability and quality levels to allow for normal values of body weight and condition.

## Introduction 

The "Baixadeiro" horse consists of a breed of native horses, typical of the Baixada Maranhense region, bred in the wilderness and acknowledged for their rusticity, physical strength as workhorses and resistant to dwelling on vast flat areas of flooded fields during the rainy season and cracked soil in the dry period. This racial group is small, with a predominant light gray or chestnut coats resulting from crossbreeding breeds introduced from the Iberian Peninsula (Gazolla et al., 2009). They are also more resistant to local environmental conditions than other breeds (Serra, 2004).

Data on the origin, reproductive characteristics and calving time of this genetic grouping are not yet known. Reports from breeders indicate that mares can give birth throughout the year. However, these horses are generally classified as seasonal breeders, presenting several cycles throughout the reproductive season. Several factors can influence reproduction, such as those related to the immune system and environmental factors such as photoperiod and temperature, nutritional factors, and seasonality (Klein and Nelson, 1999; Nagy et al., 2000, Ferreira-Dias et al., 2005).

In mares the interovulatory interval lasts an average of 21 days, varying according to the duration of estrus, a wide variation due to differences in estrus length (Cuervo-Arango et al., 2015). The growth and development of follicles occur at this stage (Moura, 2012), resulting in the dominance of a single preovulatory follicle (Morel et al., 2010). This process ends in ovulation with the formation of the corpus luteum (Arruda et al., 2001), increasing progesterone's secretion, the hormone involved in maintaining ovarian activity (Ferreira-Dias et al., 2005). In high-latitude regions, during the anovulatory season, most mares maintain progesterone levels below 1 ng / mL (Ginther, 1990), the latter being an efficient way to check the mares' ovarian cyclicity and CL activity (Arruda et al., 2001).

Traits related to follicular development may vary between breeds and between animals from different regions (Valle et al., 2005), especially those close to the equator. Information on reproductive seasonality and ovarian activity is limited (Boeta et al., 2006). In equatorial areas, seasonality of main factors affecting the success of breeding, such as the peak of grass growth, may differ from temperate areas (Carranza et al., 2017).

Estrus synchronization in equine females is a biotechnique that presents some obstacles due to the characteristics physiological of the species, however it allows manipulating the estrous cycle and follow the follicular development until the detection of ovulation. The duration of the interval between ovulations in the equine species is approximately 22 days, during which two-thirds are constituted by the luteal phase (diestrus) and one third by the follicular phase (estrus) (McKinnon and Voss, 1992).

The PGF 2α starts the regression of the corpus luteum, approximately 14 days after ovulation, in the absence of pregnancy, in large domestic species (Stabenfeldt and Edqvist, 1996). Thus, synchronization protocols that use this hormone aim to induce luteolysis of a corpus luteum and recruit a new follicular wave (Haetinger et al., 2008). The aim of this study was to evaluate the effect of synchronization with prostaglandin F2α in Baixadeiro mares during the rainy and dry seasons.

## Materials and methods

### Location and trial period

The experiment was carried out in two stages, the first during the rainy (RS - February) and dry (DS - July) 2015 season. The averages for the entire period of rainfall was 0.319 ± 1.71 mm, temperature was of 26.99 ± 2.33 °C and humidity was 84.50 ± 10.00%.

### Experimental grouping

The experiment involved fourteen mares whose mean age and mean height were, respectively, 6.35 ± 1.21 years and 128.43 ± 5.74 cm. They were originally free-ranged in the fields of the Baixada Maranhense region and introduced to the Experimental Farm of UEMA and included nulliparous, lactating and multiparous mares, all empty, evaluated by ultrasonography. From this total, nine mares were selected whose temperament and restraint allowed the blood to be collected for the hormonal assay. The animals were raised on pastures of native vegetation (*Paratheria prostata, Paspalum virgatum, and Acrocera zizanoides*; Serra, 2004), in paddocks of 0.5 ha and supplemented with commercial ration (Equimax® 12MA).

Measurements of live weight variations at 10-day intervals were recorded using equine weighing tapes and the body condition score (BCS), based on the Henneke et al., (1983) scale, considering the beginning, middle, and end of each step, to calculate the mean values per period.

### Synchronization

In both wet and dry seasons, mares were synchronized with prostaglandin F2α (Sincrocio®) to complete the previous luteal phase and control ovulation time (Faria and Gradela, 2010). For this, two doses of 1 mL of PGF2α were used with an interval of 10 days. 72 hours after the second dose, the mares were monitored by transrectal palpation and ultrasonography (Mindray® DP 2200 VET, 5 MHz transrectal transducers and 7.5 MHz frequency) for characterization of uterine edema, follicular growth and ovulation. The synchronization interval was considered the interval between the days of application of the second dose of PGF 2α and the detection of ovulation, obtaining the average of the days for mares that ovulated.

### Gynecological evaluation

By transrectal palpation, uterine tonus was classified subjectively on a scale ranging from 1 for minimal uterine tone (flaccid) to 4 for maximum tone (turgid), according to the criteria used by Hayes et al. (1985).

### Ultrasound evaluation

The ultrasound examination was performed every 48 hours to monitor the follicles that were identified and measured from static images and obtained the mean value for determining the follicular diameters. The total number of follicles was evaluated on days 0, 7 and 14 of the evaluation in which D0 was the first day of evaluation and considering the follicular emergence from the smallest follicle detected with the follicular wave involving 7 -11 follicles (Ginther et al., 2001). The classification of the follicles was based on the follicular diameter, being classified as small (≤ 15 mm), medium (16 - 30 mm) and large (≥ 30 mm; Boeta et al., 2006). Mares were considered in estrus when they had 25 mm follicles (Pierson, 1993) and were exposed to the presence of a stallion to verify sexual receptivity. The daily follicular growth rate was estimated for the dominant follicles (Almeida et al., 2001), obtaining the mean value for RS and DS. The dominant follicle was considered to be that follicle with a diameter ≥ 22.5 mm (Ginther et al., 2003). For pre-ovulatory follicle, it was considered the one with a diameter of 30 mm (Valle et al., 2005), with the pre-ovulatory period being the time elapsed between the detection of the pre-ovulatory follicle and the recording of ovulation, when the evaluations ultrasounds have become daily. The ovulation rate was determined by the frequency of ovulations recorded in both periods. After ovulation, the formation of the corpus luteum was monitored with ultrasonographic evaluations taking place at an interval of 48 hours. The interovulatory interval was considered the period between an ovulation and the subsequent ovulation.

The uterine echotexture evaluation was used to estimate the degree of the uterine score, following the Samper and Pycock ranking (Samper and Pycock, 2007), which establishes a degree of edema between 0 and 5, where 0 corresponds to the uterus without edema, 4 with strong edema and 5 with abnormal edema.

### Hormonal Assay

Blood samples collected by jugular vein puncture and stored in 5 mL heparinized tubes.

The samples were centrifuged at 1500 x g for 10 minutes, and the plasma was decanted and placed in storage tubes at -20°C (Ginther et al., 2008). Blood sampling started 72 hours after synchronization and occurred on alternate days up to 10 days after ovulation, following the days of ultrasonographic evaluation.

The levels of progesterone (P4) were determined by the enzyme immunoassay (ELISA) method, according to the procedure recommended by the commercial kit (DRG Progesterone Enzyme Immunoassay Kit, EIA - 1561, DRG - Germany) and performed at the Immunodiagnostic Laboratory of UEMA. The P4 reference solutions used in the trial were: Calibrator 0: S0 ng / mL; S1: 0.3 ng / mL; S2: 1.25 ng / mL, S3: 2.5 ng / mL, S4: 5 ng / mL; S5: 15 ng / mL; S6: 20 ng / mL. The intra-assay coefficient of variation was 5.4% for 0.62 ng/mL; 6.99% to 4.67 ng / mL; 6.86% to 10.80 ng / mL. The inter-assay coefficient of variation was 9.96% for 0.56 ng / mL; 4.34% to 4.55 ng / mL; 5.59% to 10.65 ng / mL. The readings were performed by spectrophotometer (Biotek EL x 800), the Gen5 program Getting Started, Microplate Data Collection and Analysis Software.

### Experimental design and statistical analysis

Data were analyzed by mean, standard deviation, and percentage frequency for each variable. The SAS statistical software (Student Newman Keuls test) was used to determine differences among data on weight, ECC, tonus, synchronization, rainfall, daytime duration, follicular development, and mare hormonal levels between RS and DS for animals before and after ovulation.

Data were normalized by the Cramer-von Mises test to classify follicular diameters and the analyses of treatment interactions, interactions between periods, and its relation to progesterone levels were performed through a multiple-comparison factorial using the Tukey-Kramer test. The difference in ovulation rate and number of follicles between the RS and DS periods was determined by the Chi-square test using the significance level *P* < 0.05.

### Ethics statement

The experiment was conducted at the State University of Maranhão’s Experimental Farm - Fazenda Escola de São Bento, FESB/UEMA (Lat 18°59'S; Long 56°39'W), in year 2015 and in compliance with the Ethics Commission on Animal Research (CEEA – CONCEA / MCT UEMA) approval protocol for this research work (019 / 2015).

## Results

**Figure 1:**
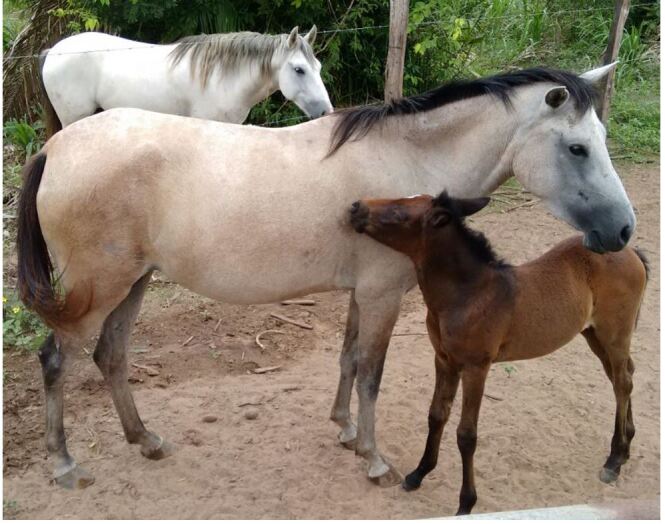
Mare and foal, behind a stalion Baixadeiro.

The mares showed no significant difference (*P* > 0.05) for weight, BSC, tonus, and uterine edema during rainy (RS) and dry (DS) periods. Mean values for each trait during RS and DS, respectively were as follows: Weight 236 kg ± 34.72 kg and 234 ± 30.78; BSC 3.60 ± 0.59 and 3.63 ± 0.58; Tonus 2.22 ± 0.22 and 2.24 ± 0.36 and Edema 0.39 ± 0.33 and 0.51 ± 0.44. The mares that had the highest weight and the highest body condition score developed large ovarian follicles. Ovulation was registered in 57.14% (8/14) of the mares in the RS and 25.57% (4/14) in the DS and the frequency of ovulation, no difference (*P* > 0.05) was observed between seasons. (Figure 1).

**Figure 2:**
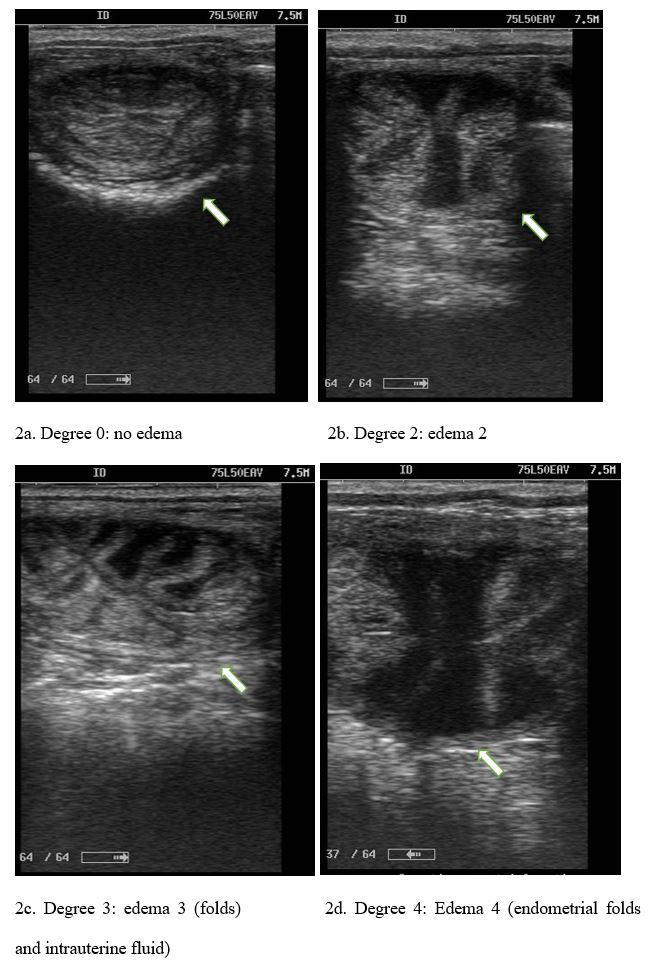
Degree of uterine edema in the rainy and dry periods ranging from 0 to 4 on the rating scale. Degree 0 = No Edema, Degree 1 = Edema, Degree 2 = Edema 2 (fluid), Degree 3 = Edema 3 (folds), Degree 4 = Edema 4 (endometrial folds and intrauterine fluid).

As for tonus and edema, changes were observed throughout the estrus, in which ovulating mares showed minimal tonus and a higher degree of edematous close to ovulation (Figure 2).

**Figure 3:**
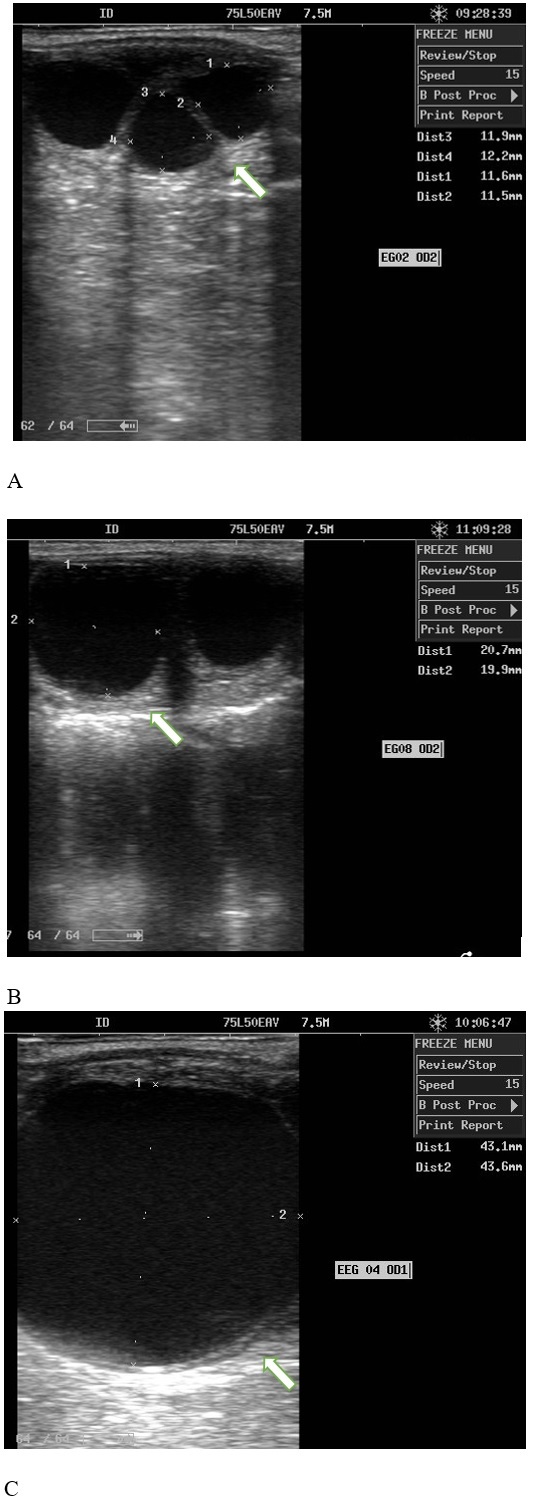
Follicular growth in the rainy and dry periods ranging. A: Small follicles < 15mm, B: Medium follicles: 15 - 30 mm, C: Large follicles > 30 mm.

There was no statistical difference between RS and DS for the total number of follicles between days 0 (6.57 ± 2.63 and 4.85 ± 2.13), days 7 (6.50 ± 3.28 and 6.85 ± 3.62 and day 14 (8.00 ± 2.23 and 7.28 ± 3.49), these are in agreement with the number of expected follicles during follicular wave emergence and are independent of body condition. The total number of small, medium and large follicles (Figure 3) in mares was different between RS and DS. Larger diameters were recorded for medium follicles in RS (RS 22.71 ± 0.27 and DS 19.50 ± 0.43), large follicles in turn had larger diameters in DS (RS 34.45 ± 0.55 and 38.44 ± 1.82), as shown in Table 1.

**Table 1:** Mean and standard deviation of follicle diameter of mares during the 2015 rainy and dry seasons.

***P* value**	**Season**	**Follicle diameter**		
		**≤ 15 mm**	**16 – 30 mm**	**≥ 30 mm**
(*P* < 0.05)	Rainy	10.97 ± 0.15 ^Ca^	22.71 ± 0.27 ^Ba^	34.45 ± 0.55 ^Ab^
(*P* < 0.05)	Dry	10.40 ± 0.21 ^Ca^	19.50 ± 0.43 ^Bb^	38.44 ± 1.82 ^Aa^

Means with different superscripts (A, B) within the row, (a, b) within the column are significant at *P* < 0.05.

Regarding the traits of the periovulatory period, no difference (*P* > 0.05) was observed for the synchronization interval (mean 12.25 ± 5.85 RS, 15.40 ± 10.52 DS), estrus (mean 7.18 ± 3.02 RS, 7.45 ± 3.05 DS) and follicular growth rate between seasons (mean 2.34 ± 0.49 RS, 1.66 ± 1.06 DS).

The analysis of follicular divergence showed no differences between the dominant follicles (RS 21.8 ± 2.00 mm / DS 21.57 ± 1.70 mm) and subordinate follicles (RS 19.03 ± 2.45 mm / DS 17.71 ± 1.90 mm) between seasons.

Differences in the preovulatory follicle diameter (*P* < 0.05 - RS 35.40 ± 2.37, DS 39.70 ± 2.77) were observed during the dry season since mares required longer periods to ovulate during this season. The maximum diameter of the preovulatory follicle was recorded on the day before ovulation and showed differences between rainy and dry periods (*P* < 0.05 - RS 35.40 ± 2.37, DS 39.70 ± 2.77), in that this difference observed was greater during the dry season, since the mares needed longer periods to ovulate in this season.

Mares showed differences (*P* < 0.05) on the mean levels of P4 between the pre and post ovulatory phases in both seasons (RS and DS), being less than 1 ng/mL before ovulation and immediately increasing its value. Differences between seasons (*P* < 0.05) were recorded in the post-ovulatory phase, when the higher the ovulation rate, the higher the DS concentration (Table 2).

**Table 2:** Mean and standard deviation of progesterone levels during the pre- and post-ovulatory phases of mares of the Baixadeiro genetic group during the 2015 rainy and dry seasons.

***P* value**	**Season**	**Progesterone levels (ng / mL)**	
		**Preovulatory**	**Post ovulatory**
(*P* < 0.05)	Rainy	0.385 ± 0.689 ^Ab^	13.993 ± 11.934 ^Aa^
(*P* < 0.05)	Dry	0.646 ± 1.416 ^Ab^	8.045 ± 6.036 ^Ba^

Means with different superscripts (A, B) within the row, (a, b) within the column are significant at *P* < 0.05.

Regardless of the season, P4 levels differed (*P* < 0.05) between the pre- and post-ovulatory phases. Climate variables differed between seasons, with daily luminosity values peaking in February when there was a difference of 12 minutes (*P* < 0.05). Total rainfall was also higher in February (Table 3), although not different.

**Table 3:** Mean and standard deviation for daily rainfall precipitation and luminosity during the experimental period.

***P* value**		**Season**	
		**Rainy**	**Dry**
(*P* < 0.05)	Daily precipitation*	4.07 ± 0.77 ^A^	1.10 ± 2.65 ^A^
(*P* < 0.05)	Daily luminosity**	12.18 ± 0.02 ^A^	11.98 ± 0.01 ^B^

Sources: *NUGEO. **INMET (2015). Means with different superscripts (A, B) within the row, (a, b) within the column are significant at *P* < 0.05.

The mares of the Baixadeiro group remained cyclical between seasons (Table 4), thus deconstructing the reproductive seasonality.

**Table 4:** Mean and standard deviation of follicular diameters and progesterone levels during the pre- and post-ovulatory phases of mares of the Baixadeiro genetic group during the 2015 rainy and dry seasons.

***P* value**	**Season**	**FOLLICULAR ACTIVITY**							
		**Pre ovulatory**			**Post ovulatory**			**P4 levels (ng / mL)**	
		**<15 mm**	**16 - 30 mm**	**> 30 mm**	**<15 mm**	**16 - 30 mm**	**> 30 mm**	**Pre**	**Post**
*P* < 0.05	Rainy	10.12 ± 4.31 ^Ca^	19.16 ± 2.84 ^Ba^	35.35 ± 3.10 ^Aa^	8.60 ± 2.97 ^Ca^	19.87 ± 3.33 ^Ba^	32.87± 1.85 ^Aa^	0.29 ± 0.54 ^Ed^	13.99 ± 11.93 ^Dd^
*P* < 0.05	Dry	10.66 ± 2.68 ^Ca^	21.21 ± 3.61 ^Ba^	39.60 ± 5.30 ^Ab^	8.08 ± 2.54 ^Ca^	18.24 ± 3.07 ^Ba^	_	0.64 ± 1.40 ^Ed^	8.04 ± 6.07 ^De^

Means with different superscripts (A, B) within the row, (a, b) within the column are significant at *P* < 0.05.

## Discussion

Horse reproduction tends to be seasonal (Nagy et al., 2000). The main adjusting factor in their original temperate ranges is photoperiod variation. However, it is absent in equatorial because, in low tropical latitudes, day length variation is minimal, and rainfall variation makes the seasonal cycle less predictable (Boeta et al., 2006; Ramírez et al., 2010; Nascimento, 2014; Ogutu et al., 2015; Carranza et al., 2017). Hence, factors affecting mares’ conditions and foaling success may influence the dates of reproduction in these areas.

Changes in tonicity during the reproductive cycle in mares have already been described (Hayes and Ginther, 1986; Cuervo-Arango et al., 2020), as the luteal phase approaches (Griffin et al., 1992; Alonso et al., 2019), with no change occurring during the anovulatory seasonality (Hayes and Ginther, 1986). Also, changes in uterine consistency and texture have been shown to occur gradually throughout estrus (Hayes et al., 1985), characterizing the estrus edema (Moura, 2012).

Our results have not evidence significant differences for weight, BSC, tonus, and uterine edema during rainy (RS) and dry (DS) periods, but mares showing ovarian activity were those with higher weight and body condition. One possible explanation for results evidenced here is that mares increase body condition after the forage grows during the rainy season and then start breeding (i.e., increase their pregnancy rate and hence foaling occurs close to this season next year). Mares largely rely on stored reserves and need to be in good condition before starting reproduction (Houston et al., 2007). However, mares with low body condition may produce fewer follicles before their first ovulation during the reproductive season (Gastal et al., 2004).

For estrous synchronization and induction, PGF 2α can be applied at any stage of the estrous cycle in two doses (Irvine, 1993). The results for synchronization intervals were higher than those reported in the literature because mares synchronized with PGF 2α ovulate between 8 - 10 days after induction (Samper, 2008), which may vary between breeds (Griffin et al., 1992; Cuervo-Arango et al., 2015), depending on the dosage administered (Cuervo-Arango et al., 2015) and follicle size at the time of administration (Samper, 2008; Bender et al., 2014).

The estrus period lasted as expected for the species, which may comprise an interval between 2 to 12 days, typically longer at the beginning of the reproductive season (Blanchard et al., 2003). These results are in agreement with those reported for marshy mares (Zúccari et al., 2002), crossbred mares (Valle et al., 2005), Purebred Arabian and Arabian Crossbred mares (Romano et al., 1998), clearly showing the variation between races of different regions, management, and environmental conditions.

The average follicle size of ovulating mares ranges from 40 to 45 mm (Blanchard et al., 2003) and can vary among breeds. In Thoroughbred mares, follicle size can range between 39.95 ± 4.84 mm (Morel et al., 2010), for the Colombian Paso Fino mares 41.34 ± 2.14 mm (Ramírez et al., 2010), with seasonal variations ranging between 48.7 ± 9.7 mm and 42.5 ± 9.1 mm (Nascimento, 2014) and for Pantaneira mares 49.5 ± 2.0 mm (Zúccari et al., 2002). For parous mares, mean follicle size is 51.7 mm, and 49.3 mm for empty mares of the Campolina Breed (Zúccari, 1990) and 34.21 ± 0.37 mm Mangalarga Marchador mares (Rodrigues et al., 2011). The range of preovulatory follicle size values emphasizes its relation to season, breed, and mare type (Ginther, 1995).

In the luteal phase, the maximum peripheral concentration of P4 is variable among mares (Carnevale et al., 1997), due to the secretory capacity of *corpus luteum* (CL) or the progesterone catabolism rate, since the factors that determine its level in cyclic mares are the cycling date and several ovulations (Nagy et al., 2004). Mature CL can produce, on average, 8 - 10 ng / mL until it undergoes luteolysis (Arruda et al., 2001) or produces values similar (Vivo et al., 1986) to those found in this study.

Concerning the follicular activity during the pre- and post-ovulatory phases, P4 levels were lower than 1 ng / mL in the presence of large and medium-sized follicles, with levels increasing after ovulation. This is because although a pool of small follicles begins to increase until ovulation, its number does not change in the ovaries throughout the estrous cycle, and what happens is that some follicles change of category (Pierson and Ginther, 1987). In the follicular phase, minimum progesterone levels can be detected, accompanied by an increase in follicular diameter during estrus and ovulation (Ginther et al., 2007). This is because changes in plasma P4 levels occur immediately after ovulation (Ginther et al., 2008), reaching 3 ng / mL (Ginther and Santos, 2015), increasing to D6 after which they plateau (Arruda et al., 2001). Similar results were reported for Arabian mares (Abo-El maaty and El-Shahat, 2012), in which the P4 serum levels were significantly higher in the luteal phase of the estrous cycle.

For these animals, weight can be considered the main factor interfering with follicular development and the interval between reproductive seasons, as BSC is associated with an increase in reproductive activity (Nagy et al., 2000; Lopes et al., 2017), as recorded for the animals from this work. Rainfall influenced the ovarian activity since most ovulation occurred during the rainy season, when pasture growth was exuberant, which promoted forage intake resulting in higher weight gain and BSC levels. The dry season at the Baixada Maranhense region sharply decreases forage availability, leading to the loss of nutritional quality, and consequently, live weight loss in equines (Santos, 1997; Barros et al., 2019). Since animals in tropical environments do not observe reproductive seasonality, when subjected to nutritional stress, mares tend to decrease their reproductive activity (Ramírez et al., 2010).

## Conclusion

Mares from the Baixadeiro genetic group did not show reproductive seasonality in conditions such as their natural environment, although there was a difference in luminosity between the rainy and dry seasons. During the pre- and post-ovulatory phases, progesterone concentrations were within the expected levels for this species, regardless of the season. The treatment with two doses of PGF 2α was effective in synchronizing the mares, promoting the return of estrus in the dry and rainy periods. Thus, mares exhibited a cyclical behavior throughout the year, exhibiting reproductive activity under conditions of adequate weight and body condition score.
